# The Rating of Perceived Exertion—Pediatric (RPE-P) Scale: Preliminary Validation

**DOI:** 10.3390/children10121906

**Published:** 2023-12-10

**Authors:** Brynn LiaBraaten, Stacy Stolzman, Pippa M. Simpson, Liyun Zhang, Taylor Brockman, Nina Linneman, Steven J. Weisman, Keri R. Hainsworth

**Affiliations:** 1 Department of Anesthesiology, Medical College of Wisconsin, Milwaukee, WI 53226, USA; bliabraaten@mcw.edu (B.L.); sweisman@mcw.edu (S.J.W.); 2 Jane B. Pettit Pain and Headache Center, Children’s Wisconsin, Milwaukee, WI 53226, USA; 3 Department of Physical Therapy, Concordia University Wisconsin, Mequon, WI 53097, USA; 4 Division of Quantitative Health Sciences, Medical College of Wisconsin, Milwaukee, WI 53226, USA

**Keywords:** validation, pain, weight, perceived exertion, exercise, pediatrics

## Abstract

Physical activity is critical to functional rehabilitation for youth with chronic pain, which may be especially true for those with co-occurring obesity. To facilitate the development of physical activity interventions for youth with chronic pain, the newly developed “Rating of Perceived Exertion—Pediatric” scale was modeled after the widely used pain numeric rating scale-11. This study is an initial evaluation of the scale in a sample of adolescents (*n* = 157, 13–17 years, 51% female) with four subgroups: (1) healthy controls (healthy weight/no pain); (2) chronic pain/healthy weight; (3) obese (no pain); (4) chronic pain/obese. Participants rated perceived exertion using the new scale and the Borg 6–20 Scale of Perceived Exertion while holding a three-minute yoga pose (Warrior II). In the whole sample, the Perceived Exertion—Pediatric scale showed good concurrent (*p* < 0.001), convergent (all *p*s < 0.05), discriminant (*p* = 0.431), and known-groups validity (all *p*s < 0.05). The chronic pain subgroup also showed good concurrent (*p* < 0.001), mixed convergent (*p*s < 0.001 to 0.315), and good discriminant validity (*p* = 0.607). Limitations include the restricted age range, lack of diversity, and lack of test-retest reliability. The RPE-P shows promise as an assessment tool for perceived exertion in adolescents with and without chronic pain.

## 1. Introduction

Physical activity is critical for youth with chronic pain [1] because of its ability to reduce pain and systemic inflammation, as well as improve mood and sleep [2]. However, increasing physical activity in youth with chronic pain is not without complex concerns. It can increase pain [3], something about which youth with chronic pain are often fearful [4]. This fear of general pain and of pain associated with activity can lead to inconsistent or limited physical activity participation or avoidance altogether [3,4]. Therefore, physical activity recommendations for youth with chronic pain must be made thoughtfully [1]. As a compounding factor, up to 68% of those presenting to pediatric pain clinics either have or are considered at risk of developing obesity [5]. This makes it particularly important to tailor physical activity recommendations not only to a patient’s pain level, but to other factors that may limit physical abilities, such as excess weight [6]. One way to develop appropriate physical activity interventions for youth with chronic pain is to account for each patient’s fitness level, training load, and exercise intensity through the assessment of perceived exertion [7,8,9,10]. Unfortunately, there are no existing pediatric scales validated for youth with chronic pain. To meet the need for a perceived exertion scale for youth that can also be used for youth with chronic pain, we developed the “Rating of Perceived Exertion—Pediatric Scale” (RPE-P). The purpose of this study was to evaluate the initial validity of the RPE-P in a group of adolescents.

The construct of perceived exertion is defined as the degree of effort or strain during activity [7]. In adults, ratings of perceived exertion have shown considerable utility and practicality in exertion estimations, exercise prescriptions, and regulation of exercise intensity [11]. In contrast, few studies exist on ratings of perceived exertion for youths [11]. These studies have been limited by the types of exercise used (primarily focusing on aerobic exercise, such as running [12,13], biking [14,15], and soccer [16,17]), small [18,19,20] and selective sample sizes [12,19,21], and restricted age ranges [16,22]. Although the Borg 6–20 Category Scale [23] and the Borg Category-Ratio-10 Scale [23] are among the scales most widely utilized to rate perceived exertion, a scoping review of pediatric studies [11] found that the Borg scales show inconsistent validity across studies, bringing into question their use in pediatrics [11,24]. There have been a variety of pediatric pain scales developed, but research has shown that even minimal alterations to versions of these scales (e.g., orientation of the scale’s line or labels) can significantly affect scale validity [25]. With regard to existing perceived exertion scales, the use of non-horizontal scales with or without images [26,27,28,29] may make them less than ideal for use alongside pain intensity scales for youth with chronic pain.

For pediatric patients with chronic pain, their pain impacts all aspects of life, but perhaps most concerning is the impact on the ability to participate in everyday activities, a construct referred to as functional disability [26,27]. Estimates indicate that after the development of chronic pain, up to 75% of youth reduce both rigorous sports activities as well as routine/leisure activities [30]. Additionally, research supports that youth with co-occurring chronic pain and obesity have impaired physical functioning, even beyond that associated with chronic pain alone [31]. It is plausible that the lower levels of physical activity and impairments in physical functioning may translate into greater ratings of perceived exertion for youth with chronic pain, regardless of their weight status. This suggests that a valid scale for determining perceived exertion is not only warranted but could be vitally important to the field of pediatric pain.

The primary aim of the current study was to use an isometric yoga pose to validate the Rating of Perceived Exertion—Pediatric Scale (RPE-P) in an adolescent sample. Our secondary aim was to examine the validity of the RPE-P in the participants with chronic pain, regardless of their weight status. We hypothesized that the RPE-P scale would show (1) concurrent validity by examining the relationship between RPE-P and concurrent ratings of perceived exertion using the Borg 6–20 Category Scale (hereafter, “Borg”); (2) convergent validity, by examining associations between the RPE-P and ratings of concurrent muscle pain, current pain intensity, fear of pain, and sleep quality; (3) discriminant validity, by examining the relationship between RPE-P ratings and ratings of meal nutritional quality; and (4) cross-sectional construct validity used to establish known-groups validity, by examining between-group differences in ratings of perceived exertion for both the RPE-P and Borg scales [32]. For known-groups validity, we hypothesized that youth with chronic pain alone (without obesity) would report higher ratings of perceived exertion than youth in the healthy control group, and that the chronic pain with obesity subgroup would report higher ratings of perceived exertion than all other groups. Additionally, we examined the ratings of perceived exertion over time, with the expectation that both of the chronic pain groups may show ratings of perceived exertion that increase faster than ratings of perceived exertion reported by youth with obesity without chronic pain, or healthy controls.

## 2. Materials and Methods

### 2.1. Participants

As part of a larger study (currently unpublished), four groups were recruited according to presence/absence of obesity and chronic pain: (1) Healthy Controls (HC; healthy weight with no pain); (2) Chronic Pain with Healthy Weight (CPHW); (3) Obese (O; no pain); and (4) Chronic Pain with Obesity (CPO). Participants with chronic pain were recruited from intake appointments in a multi-disciplinary pain and headache clinic (CPHW and CPO groups) in the Midwestern United States. Participants without chronic pain were recruited from a pediatrician’s office affiliated with the children’s hospital (O and CPO groups).

Inclusion and exclusion criteria are detailed in a prior study [33]. Body mass index (BMI) percentiles were based on age and sex [34]. Healthy weight was defined as having a BMI ≥ 5th and <85th percentile and obesity was defined as a BMI ≥ 95th percentile. This study was conducted from May 2018 through January 2020. The hospital Institutional Review Board (IRB) approved the use of these data for study purposes on 3 January 2018. Participants and parents gave written assent and consent to participate, respectively. Participants received a gift card as compensation for their time, and the parents received a gas card.

### 2.2. Procedure

The study took place in the Pediatric Translational Research Unit of the hospital. Aspects of the protocol relevant to this study included the following elements in the following order:

(1) Familiarization to ratings of perceived exertion and muscle pain scales.
(2) Paper and pencil questionnaires (described below).
(3) Yoga pose (Warrior II) held for three, 1 min iterations (with a 30 s break after minutes 1 and 2).

Participants were shown three scales (RPE-P, Borg, and muscle pain) on a large (29.5 in. × 19 in.) foam board. The principal investigator (KH) explained the meaning of each scale, the meaning of the respective ratings, and the range of response options for each scale. This was followed by completion of paper and pencil questionnaires. The principal investigator then explained and demonstrated the yoga pose, utilizing a pre-established script. While demonstrating the pose, the principal investigator also demonstrated how participants would call out the three ratings (one for each scale) during each 1 min iteration of the pose.

The yoga pose selected for the study was Warrior II. Given the possibility that some participants might have difficulty holding the pose for 3 consecutive minutes and/or have balance difficulties, the pose was modified in the following ways: First, participants were given a 30 s break (participant stood comfortably in place with arms dropped and legs straight) after minute 1 and after minute 2. Additionally, one side of the body (outside of foot and hand/arm) was pressed against a wall to increase stability during the pose. Participants had a yoga strap wrapped around the stable arm with the end of the strap held tautly in the extended hand to increase effort in the extended arm. A certified Iyengar yoga teacher with 9 years of experience trained the principal investigator to perform the pose and worked with the principal investigator to develop scripted instructions to use when teaching each participant how to perform the pose (available upon request). The teacher trained the principal investigator to 100% criterion over 3 separate sessions (including watching/instructing the principal investigator as she taught volunteers, to ensure that the method of instruction was correct and was consistently applied across participants).

During each minute of the pose, participants rated perceived exertion using the RPE-P and the Borg Scale of Perceived Exertion, along with ratings of muscle pain. The presentation order of the RPE-P and Borg was counter-balanced across participants (always appearing as first or second scales), with the muscle pain scale always assessed third. All scales were visible to participants (shown at eye level in front of participants’ gaze) throughout the duration of the iterations of the yoga poses. The participants verbally called out ratings for each of these scales two times during each of the three iterations of the yoga pose. This totaled to two ratings per minute, and six total ratings across the three 1 min iterations of the yoga pose. A study team member verbally prompted participants to call out the six ratings two times during each minute of the pose (at 30 and 55 s after the start of each minute of the pose).

### 2.3. Measures

#### 2.3.1. Rating of Perceived Exertion—Pediatric (RPE-P) Scale

The RPE-P scale is a single-item scale measuring perceived exertion. We modeled this scale after the Numeric Rating Scale-11 (NRS-11) for pain [35,36]. Participants were shown the question, “How hard are you working?” with a horizontal scale ranging from 0 (“extremely easy”) to 10 (“extremely hard”). Anchors were the same as those used in an existing pediatric perceived exertion scale, the OMNI scale of perceived exertion. The anchors were shown under every even number (0, not tired at all; 2, a little tired; 4, getting more tired; 6, tired; 8, really tired; 10, very, very tired) [29].

#### 2.3.2. Borg Scale 6–20 Category Scale of Perceived Exertion

The Borg Scale of Perceived Exertion is a single-item perceived exertion scale ranging from 6 (“no exertion”) to 20 (“maximal exertion”). Participants were shown the question, “How hard are you working” along with a vertical scale. The scale ranges from 6 to 20 with descriptive anchors of physical effort next to over half the numbers. The Borg scale has shown to have a reliability coefficient of 0.78 [37]. This measure has inconsistent validation in adolescents and pre-pubescent children [24].

#### 2.3.3. Ratings of Concurrent Muscle Pain

The NRS-11 for pain was modified for this study. The pain scale used in this study consisted of a horizontal line of numbers (consistent with a NRS for pain), with anchors below even numbers. The anchors were the same as those used in the Children’s OMNI scale of muscle hurt [28]. Participants were instructed to call out the intensity of their muscle pain (0 = do not hurt, 2 = hurt a little, 4 = hurt more than a little, 6 = hurt even more, 8 = hurt a lot, 10 = hurt worst) during the yoga pose.

#### 2.3.4. Physical Activity Questionnaire for Adolescents (PAQ-A)

The Physical Activity Questionnaire is a 10-item self-report tool utilized for assessing a youth’s involvement in activities and sports in the previous 7 days. Higher PAQ-A scores represent more physical activity. The PAQ-A is designed for adolescents aged between 14–20 and is reliable and valid [38].

#### 2.3.5. Current Pain

Patients rated their current pain using a numerical rating scale for pain. Participants were shown a horizontal line of numbers from 0 (no pain) to 10 (worst pain possible) and were asked to circle the number corresponding to their current pain intensity.

#### 2.3.6. Fear of Pain Questionnaire-Child (FOPQ-C)

The FOPQ-C is a self-report questionnaire of pain-related fear. This measure has 24 items to assess a total score. A total of 13 of these items assess the Fear of Pain subscale, and 11 of these items assess the Avoidance of Activities subscale. All items are rated from 0 = strongly disagree to 4 = strongly agree. Higher scores indicate higher levels of pain-related fear. As reported in the initial validation study, internal consistency is good (alpha 0.89 for Fear of Pain and 0.87 for Avoidance of Activities) [4].

#### 2.3.7. Adolescent Sleep-Wake Scale—Short Form (ASWS)

The ASWS is a 10-item measure of sleep quality in the previous month, including insomnia. Items assess sleep in 5 behavioral dimensions: going to bed, falling asleep, maintaining sleep, reinitiating sleep, and return to wakefulness. Lower scores indicate lower sleep quality. The short form was validated in a pediatric pain sample [39].

#### 2.3.8. Meal Nutritional Quality

Questions about the type of foods served at family meals were used to assess meal nutrition. They were developed for the University of Minnesota Project F-EAT (Families and Eating and Activity Among Teens) [40]. Six items were specifically designed to measure the types of food served at family meals, for example “Think about a typical family dinner at your home. Is a green salad served?”. All items are rated on a 4-point scale ranging from 1 = never to 4 = always. One item was reverse scored (Are sugar sweetened beverages (soda pop, Kool-aid, etc.) served?). Meal nutrition total scores range from 6–24, with higher numbers indicating healthier nutrition. The items have shown good reliability [40].

### 2.4. Statistical Analyses

All analyses (with the exception of the known-groups analyses described below) are presented for the whole sample (*N*= 157) and for the chronic pain subgroup (i.e., combined subgroups of youth with chronic pain with or without obesity, CP and CPO groups, *n* = 77). Data were checked for normality via the Kolmogorov–Smirnov and Levene’s tests. Continuous variables are reported as means (±SD) and median and interquartile range, as appropriate. Categorical variables are presented as *n* (%). Between-group differences in physical activity were examined using a general linear model with Tukey post-hoc tests. To compare differences between groups, a Kruskal–Wallis test was used for continuous variables, and an χ^2^ test was used for categorical variables. The dataset had no missing data. To assess concurrent, convergent and discriminant validity, Pearson’s correlation tests were used to examine relationships between RPE-P ratings and other constructs. To examine the changes of each outcome over time and by group, the known-groups analyses were conducted using a generalized linear model with repeated measures. Pairwise comparisons were made using Least Significant Difference adjustments for multiple comparisons. A 2-sided *p*-value of 0.05 was considered statistically significant. Data were analyzed using SPSS 26.0 (IBM).

## 3. Results

### 3.1. Participants

The sample consisted of 157 adolescents ranging from 13–17 years of age (Mdn 15.00, IQR 14.00–16.00). Fifty-one percent (*n* = 80) were female, 85% (*n* = 133) were White and 86% (*n* = 135) were non-Hispanic or Latino (see Table 1). Each group consisted of 40 participants (20 female/20 male), except the CPO group (*n* = 37) with 20 female and 17 male participants. The groups were balanced in demographics (i.e., age, sex, and ethnicity; all *p*s > 0.05), with the exception of race. Black and American Native participants were slightly over-represented in the CPO group, as compared with all other groups (*p* = 0.005). As expected, based on recruitment to specific groups, participants differed on BMI (*p* < 0.001), pain type (*p* = 0.041), and number of days participants had pain in the previous two weeks (*p* < 0.001). Physical activity levels differed across groups (*p* < 0.001). Pairwise comparisons showed that the HC group reported higher levels of physical activity than the CPHW group (*p* < 0.001), O group (*p =* 0.006), and CPO group (*p* < 0.001). No differences were found between any of the other groups (all *p*s > 0.05).

### 3.2. Psychometric Properties—Whole Sample

**Figure 1:**
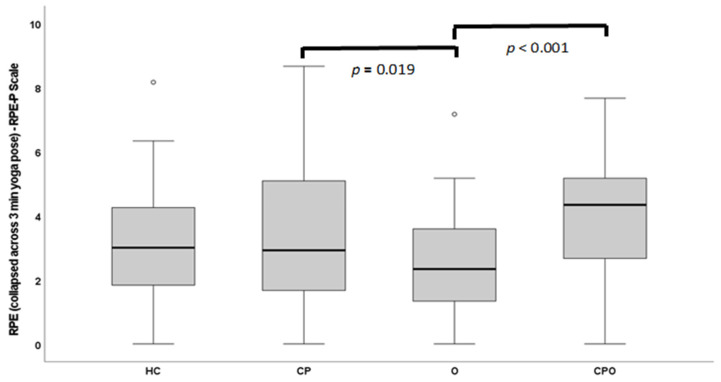
RPE-P ratings collapsed across the 3-min yoga pose.

**Figure 2:**
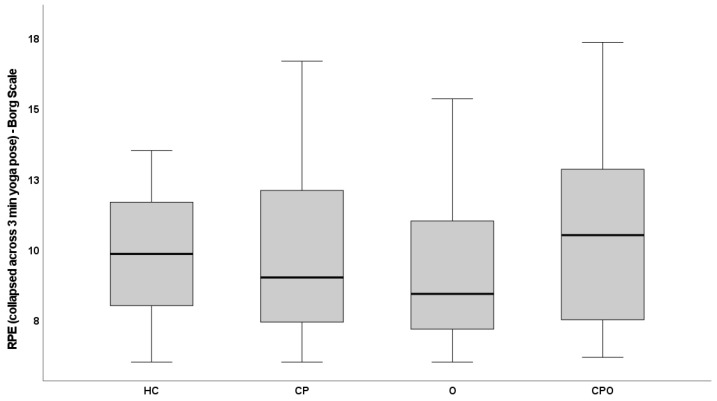
Borg ratings collapsed across the 3-min yoga pose.

Whole sample: For the whole sample, the median and IQR of participants’ average ratings of perceived exertion (collapsed across all three minutes) was 3.00 (1.75–4.67), with a range of 0 to 10. Within individual minutes of the yoga pose, RPE-P scores ranged from 0–8 in minute 1, 0–8.5 in minute 2, and 0–10 in minute 3. See Figure 1 and Figure 2 for perceived exertion ratings across minutes of the yoga pose.

Chronic pain subgroup: For the chronic pain subgroup, the median and IQR of participants’ average ratings of perceived exertion (collapsed across all three minutes) was 3.67 (2.08–5.17), with a range of 0–10. Within minutes of the yoga pose, RPE-P scores ranged from 0–8 in minute 1, 0–8.5 in minute 2, and 0–10 in minute 3, the same ranges as the whole sample.

### 3.3. Concurrent Validity

Whole sample: The RPE-P showed excellent concurrent validity. RPE-P ratings were strongly correlated with ratings of perceived exertion using the Borg scale (*r* = 0.86, *p* < 0.001).

Chronic pain subgroup: The RPE-P showed excellent concurrent validity for the chronic pain subgroup. RPE-P ratings were strongly correlated with ratings of perceived exertion using the Borg scale (*r* = 0.89, *p* < 0.001).

### 3.4. Convergent Validity

See Table 2 for descriptive statistics on all measures used to evaluate convergent validity.

Whole sample: Overall, the RPE-P showed good convergent validity. RPE-P ratings were strongly correlated with concurrent muscle pain ratings (*r* = 0.89, *p* < 0.001). RPE-P ratings were also weakly but significantly correlated with current pain (*r* = 0.27, *p* < 0.001), FOPQ total scores (*r* = 0.33, *p* < 0.001), as well as FOPQ fear of pain (*r* = 0.29, *p* < 0.001), and FOPQ avoidance of activities (*r* = 0.34, *p* < 0.001) subscales. RPE-P ratings were also negatively correlated with ASWS scores (*r* = −0.19, *p* = 0.016).

Chronic pain subgroup: Overall, the RPE-P showed mixed results for convergent validity based on reports from the chronic pain subgroup. RPE-P ratings were correlated with both concurrent muscle pain ratings (*r* = 0.93, *p* < 0.001) and FOPQ avoidance of activities (*r* = 0.25, *p* = 0.029). However, RPE-P ratings were unrelated to current pain (*r* = 0.16, *p* = 0.153), FOPQ total scores (*r* = 0.20, *p* = 0.083), FOPQ fear of pain (*r* = 0.14, *p* = 0.229), and ASWS scores (*r* = −0.12, *p* = 0.315).

### 3.5. Discriminant Validity

Whole sample: The RPE-P showed good discriminant validity. RPE-P ratings were unrelated to Meal Nutrition total scores (*r* = −0.06, *p* = 0.431).

Chronic pain subgroup: The RPE-P showed good discriminant validity. RPE-P ratings were unrelated to Meal Nutrition total scores (r = −0.04, *p* = 0.607).

### 3.6. Cross-Sectional Construct Validity—Known-Groups Validity

Descriptive data for ratings of perceived exertion across time for both the RPE-P and Borg Scales are shown in Table 3.

The RPE-P showed a significant effect of time (*p* < 0.001), with ratings of perceived exertion increasing linearly over the three minutes of the pose, with minute 1 < minute 2 < minute 3 (all pairwise comparisons, *p* < 0.001). The RPE-P also showed a significant group effect (*p* = 0.006) but no time × group interaction (*p* = 0.615). Pairwise comparisons showed that the CPHW group reported significantly greater ratings of perceived exertion than the group with obesity (*p* = 0.019). In addition, the CPO group reported greater ratings of perceived exertion than the O group (*p* < 0.001). All other comparisons were not significant (*p*s > 0.050).

As with the RPE-P, the Borg showed a significant effect of time (*p* < 0.001). The ratings of perceived exertion increased linearly over the three minutes of the pose, with minute 1 < minute 2 < minute 3 (all pairwise comparisons, *p* < 0.001). In contrast to the RPE-P data, the Borg did not show a group effect (*p* = 0.102) and did not show a time × group interaction (*p* = 0.302).

## 4. Discussion

The accurate assessment of perceived exertion in youth with chronic pain may have considerable clinical utility. To that end, this study examined preliminary validity of a new scale developed to capture ratings of perceived exertion in a sample of adolescents. To establish preliminary validity for use in pediatric chronic pain specifically, we evaluated the RPE-P in a chronic pain subgroup. The main findings included demonstration of: (1) concurrent validity for the whole sample and chronic pain subgroup, (2) convergent validity for the whole sample, with mixed results for the chronic pain subgroup, (3) good discriminant validity for the whole sample and chronic pain subgroup, and (4) good cross-sectional or known-groups validity for the tool. These findings suggest that the RPE-P may be a useful tool in clinical and research applications for youth in general, and for youth with chronic pain.

For the entire sample, the RPE-P showed good convergent validity across all measures. This included positive associations between RPE-P ratings and ratings of concurrent muscle pain, current pain, Fear of Pain (FOPQ) total scores, fear of pain and avoidance of activities subscales, and a negative association with sleep quality scores. For the chronic pain subgroup, results of the convergent validity assessments were mixed. It is important to note that of the measures used for these analyses, concurrent muscle pain and avoidance of activities are among the most important, particularly in youth with chronic pain. For the chronic pain subgroup, RPE-P ratings were associated with both measures. The association between concurrent muscle pain and ratings of perceived exertion in our study is similar to an association between ratings of perceived exertion and pain immediately after exercise in a sample of adults with fibromyalgia [41]. The lack of an association between ratings of perceived exertion and the other measures may have occurred for one or more reasons. First, the analyses with the chronic pain subgroup were reduced in power and variance, as compared with the analyses based on the whole sample. It is possible that the small subsample size was a limitation in this respect. Second, the subgroup of participants with chronic pain consisted primarily of youth with chronic headache/migraine pain, which may have further reduced variability in the measures used to assess convergent validity. Future studies would benefit from larger and more diverse samples (e.g., representing more pain diagnoses). Third, obesity-related differences in the constructs used to assess convergent validity precluded the opportunity to observe convergent relationships. This limitation would only be compounded by the small subsample size. While it is plausible that youth with chronic pain with/without obesity may differ on concurrent pain, fear of pain (including the fear of pain total and subscale scores), and sleep quality, little is known about such differences. Finally, while the scale was modeled after the NRS-11 for pain, the anchors were adapted from an existing pediatric rating of perceived exertion scale [29]. It is possible that the RPE-P deviated too far from the NRS-11 for pain. The latter uses anchors only for ratings of 0 and 10, whereas the RPE-P used anchors for all even numbers (six anchors in total). Future studies should evaluate the RPE-P with other markers of exercise intensity (both in general, as well as those that may be expected to differ in youth with chronic pain).

The Borg scale is one of the most widely used scales for the assessment of ratings of perceived exertion [11]. Our data align with other reports that show that the Borg scales have inconsistent validity across pediatric studies [11,24]. In the current study, the Borg scale did not differentiate between-group differences in ratings of perceived exertion and therefore did not show evidence for known-groups validity. This was unexpected given that higher levels of perceived exertion were expected for the groups with chronic pain, and the fact that the healthy control group reported higher levels of physical activity than all other groups. For youth with chronic pain, it is important that the measure be sensitive to differences between youth with and without chronic pain. Although participants with CPO reported the highest ratings of perceived exertion, our initial hypothesis was not supported. Nonetheless, the RPE-P did show evidence for known-groups validity as participants in both the CPHW and CPO groups had significantly higher ratings of perceived exertion than participants with obesity alone (without chronic pain). This is consistent with the findings of Homann et al. in a sample of adults with fibromyalgia; the chronic pain (fibromyalgia) group showed higher ratings of perceived exertion during and after a 6-min walk test, while the control group did not [42]. Given the prevalence of childhood and adolescent obesity in chronic pain [5,43] and in the United States [34], it is vital to have a scale of perceived exertion that can differentiate between adolescents across weight categories, in turn, allowing for the accurate assessment of perceived exertion. Based on existing studies, perceived exertion ratings can vary based on a number of factors (e.g., age [44], sex [45], anticipatory factors [46], and instruction format [47]), including weight [48]. Until the RPE-P undergoes further validation, current evidence suggests that the RPE-P may perform better than the Borg if the sample has significant representation of youth with obesity.

As hypothesized, the scale showed responsiveness to expected changes in exertion over time, increasing linearly across the three minutes of the yoga pose. These results are consistent with other studies. In Stolzman et al. [49], adolescents with healthy weight and those with overweight/obesity participated in an aerobic capacity treadmill test. Results showed that after the first three minutes of the test, the youth with overweight/obesity reported higher ratings of perceived exertion than the youth with a healthy weight. Based on this and other evidence, it was expected that the CPO group would endorse the highest ratings of perceived exertion, given the known impairments in physical functioning in this group [31,50].

This preliminary evidence for the validation of the new measure may not be surprising, given the historical attempts to validate pediatric pain scales. The RPE-P was purposely modeled after the NRS-11 for pain because research has shown that even minimal changes in versions of pain scales (e.g., orientation of the scale’s line or labels) significantly affect the validity and properties of the scale [25]. The NRS-11 is widely used and well-validated in the context of pediatric pain [35,36]. To exemplify, a systematic review by Birnie et al. [51] examined 80 studies focused on self-reported pain measures in children and adolescents; the NRS-11 was one of only two measures that were recommended for all types of pain (acute pain, postoperative pain, and chronic pain) for children 8 years and older.

Study strengths and limitations: This is the first study to evaluate a scale of perceived exertion designed to consider youth with chronic pain. Other strengths include the sample size and the subgroups varying in presence/absence of chronic pain and obesity. One limitation of this study was the restricted age range (13–17 years), which limits the generalizability of findings to younger children. Future studies should evaluate the validity of the RPE-P down to 8 years, which would be similar to the age cut-off for the pain NRS-11. The sample was also primarily comprised of Caucasian (and non-Hispanic or Latino) participants, which limits the generalizability to more diverse samples. Future studies should investigate the use of the RPE-P with participants who experience different types of chronic pain and evaluate whether it is a valid measure of perceived exertion when youth are engaged in different forms of exercise (e.g., aerobic). Futures studies should also analyze the test-retest reliability, as this was not feasible within the present study. It is also known that psychological factors such as catastrophizing influence ratings of perceived exertion [52]; future studies should examine these factors and their effect. Finally, health is a multidimensional and nuanced concept, and it is known that BMI as a construct has limitations and carries stigma [53]. Future studies may consider whether experiences of stigma influence ratings of perceived exertion. Additionally, further screening beyond BMI (e.g., metabolic function, adiposity) should be considered in order to avoid some of the limitations of BMI alone as a screening index.

## 5. Conclusions

The RPE-P scale showed preliminary validity in a sample of adolescents as well as a subgroup of adolescents with chronic pain. The main findings included demonstration of: (1) concurrent validity for the whole sample and chronic pain subgroup, (2) convergent validity for the whole sample, with mixed results for the chronic pain subgroup, (3) good discriminant validity for the whole sample and chronic pain subgroup, and (4) good cross-sectional or known-groups validity for the tool. The RPE-P shows promise as an assessment tool for perceived exertion in adolescents in general and for those with chronic pain.

## Figures and Tables

**Table 1:** Participant Characteristics and Baseline Pain.

Group	Combined *N* = 157	HC *n* = 40	CPHW *n* = 40	O *n* = 40	CPO *n* = 37	*p*
Age						0.286
Mdn	15.00	15.00	15.00	14.00	15.00	
IQR	14.00–16.00	14.00–16.00	13.25–16.00	13.00–15.75	14.00–16.00	
Sex, *n* (%)						0.980
Female	80 (51.0)	20 (50.0)	20 (50.0)	20 (50.0)	20 (54.1)	
Male	77 (49.0)	20 (50.0)	20 (50.0)	20 (50.0)	17 (45.9)	
Race, *n* (%)						**0.005**
White	133 (84.7)	38 (95.0)	35 (87.5)	35 (87.5)	25 (67.6)	
Black	10 (6.4)	0 (0.0)	4 (10.0)	0 (0.0)	6 (16.2) *	
Asian/Pacific Islander	0 (0.0)	0 (0.0)	0 (0.0)	0 (0.0)	0 (0.0)	
American Native	2 (1.3)	0 (0.0)	0 (0.0)	0 (0.0)	2 (5.4) ^†^	
Native Hawaiian	0 (0.0)	0 (0.0)	0 (0.0)	0 (0.0)	0 (0.0)	
More than One Race	12 (7.6)	2 (5.0)	1 (2.5)	5 (12.5)	4 (10.8)	
Ethnicity, *n* (%)						0.191
Not Hispanic or Latino	135 (86.0)	37 (92.5)	33 (82.5)	36 (90.0)	29 (78.4)	
Hispanic or Latino	19 (12.1)	2 (5.0)	5 (12.5)	4 (10.0)	8 (21.6)	
Prefer not to Answer	3 (1.9)	1 (2.5)	2 (5.0)	0 (0.0)	0 (0.0)	
BMI Percentile						**<0.001**
Mdn	84.59	55.76	55.95	96.95	98.63	
IQR	55.40–97.40	37.12–70.65	36.85–73.20	95.26–98.32	96.37–99.52	
Physical Activity						**<0.001**
M	2.43	2.87	2.22	2.39	2.22	
SD	0.69	0.52	0.67	0.72	0.66	
Pain Type, *n* (%) ^§^		N/A ^‡^		N/A ^‡^		**0.041**
Headache/Migraine	58 (75.3)		25 (62.5)		33 (89.2)	
Trunk	5 (6.5)		4 (10.0)		1 (2.7)	
Extremities	6 (7.8)		6 (15.0)		0 (0.0)	
Abdomen	7 (9.1)		4 (10.0)		3 (8.1)	
Other	1 (1.3)		1 (2.5)		0 (0.0)	
How many days in the past 2 weeks had pain						**<0.001**
Mdn	3.00	1.00	6.00	2.00	7.00	
IQR	1.00–7.00	0.00–2.00	4.00–13.75	0.00–3.00	3.00–12.50	
Current Pain on Day of Appointment						
Mdn	0.00	0.00	2.00	0.00	2.00	
IQR	0.00–2.00	0.00–1.00	0.00–4.00	0.00–0.00	0.00–5.00	

Bolded *p* values indicate significant between-group differences. * (Std. Resid. 2.4); Black youth were over-represented in the CPO group. ^†^ (Std. Resid. 2.2); American Native youth were over-represented in the CPO group. ^‡^ These groups were not assessed for pain type as they do not have chronic pain. ^§^ Combined sample for this question, *n* = 77. HC = Healthy Control group; CPHW = Chronic Pain with Healthy Weight group; O = Obesity with no pain group; CPO = Chronic Pain with Obesity group; Mdn = Median; IQR = Inter-Quartile Range; BMI = Body Mass Index.

**Table 2:** Descriptive statistics of measures used for convergent validity.

Group	Combined *N* = 157	CP Subgroup *n* = 77
FOPQ-C Total		
M (SD)	24.03 (20.38)	37.34 (19.51)
Mdn (IQR)	22.00 (6.00–38.00)	37.00 (24.00–46.50)
FOPQ-C Avoidance		
M (SD)	11. 40 (9.91)	18.38 (8.99)
Mdn (IQR)	9.00 (2.50–18.50)	18.00 (12.00–24.00)
FOPQ-C Fear of Pain		
M (SD)	12.62 (11.47)	18.96 (11.87)
Mdn (IQR)	10.00 (3.00–20.00)	18.00 (10.00–27.00)
ASWS Total		
M (SD)	4.19 (0.82)	3.82 (0.90)
Mdn (IQR)	4.30 (3.80–4.80)	3.90 (3.10–4.45)
Current Pain		
M (SD)	1.38 (1.98)	2.40 (2.28)
Mdn (IQR)	0.00 (0.00–2.00)	2.00 (0.00–4.50)
Mean RPE-P		
M (SD)	3.26 (1.97)	3.73 (2.10)
Mdn (IQR)	3.00 (1.75–4.67)	3.67 (2.08–5.17)
Mean Borg		
M (SD)	9.75 (2.61)	10.15 (2.90)
Mdn (IQR)	9.33 (7.50–11.92)	10.00 (7.42–12.50)
Mean MP		
M (SD)	2.61 (1.90)	3.17 (2.01)
Mdn (IQR)	2.33 (1.00–3.92)	2.83 (1.75–4.75)

**Table 3:** **Descriptive data on** ratings of perceived exertion across time for the RPE-P and Borg scales.

Group	Combined *N* = 157	HC *n* = 40	CPHW *n* = 40	O *n* = 40	CPO *n* = 37
RPE-p Minute 1					
Mdn	2.50	2.50	3.00	2.00	4.0
IQR	2.0–4.0	1.6–3.5	1.3–4.9	1.3–3.0	2.0–5.0
RPE-P Minute 2					
Mdn	3.00	3.00	2.50	2.00	4.50
IQR	2.0–4.5	2.0–4.0	21.5–5.0	1.0–3.0	3.0–5.0
RPE-P Minute 3					
Mdn	3.50	3.50	3.25	3.00	4.50
IQR	2.0–5.0	2.0–5.0	2.0–5.5	1.1–4.0	3.0–6.0
RPE-P All Mins.					
Mdn	3.00	3.00	2.92	2.33	4.33
IQR	1.75–4.67	1.8–4.3	1.7–5.1	1.3–3.63	2.6–5.3
Borg Minute 1					
Mdn	8.5	8.75	8.50	8.00	10.50
IQR	7.0–11.0	7.0–10.4	7.0–11.5	7.0–9.9	7.0–12.0
Borg Minute 2					
Mdn	9.50	9.50	9.25	8.8	11.00
IQR	7.5–12.0	7.6–11.5	7.5–12.5	7.0–11.4	7.8–12.8
Borg Minute 3					
Mdn	10.50	11.00	9.00	9.25	11.50
IQR	8.0–12.5	8.6–12.5	7.1–13.4	7.0–12.0	7.8–13.8
Borg All Mins.					
Mdn	9.33	9.83	9.00	8.42	10.50
IQR	7.5–11.9	8.0–11.8	7.4–12.1	7.2–11.1	7.4–12.8

HC = Healthy Control Group; CPHW = Chronic Pain with Healthy Weight group; O = Obesity with no pain group; CPO = Chronic pain with obesity group; M = Mean; SD = Standard Deviation; Mdn = Median; IQR = Inter-Quartile Range; RPE-P = Rating of Perceived Exertion-Pediatric Scale, Borg = Borg 6–20 Category Scale.

## Data Availability

The data presented in this study are available on request from the corresponding author. The data are not publicly available due to patient privacy concerns.
